# Exploring clinician-reported assessments of capacity and performance qualifiers in the ICF: a scoping review

**DOI:** 10.3389/fresc.2026.1792865

**Published:** 2026-05-07

**Authors:** Tobias Kaarsbo, Jeppe Grabov Phillip, Thomas Maribo, Kristian Hansen, Huib Ten Napel, Lisbeth Rosenbek Minet

**Affiliations:** 1 Department of Geriatric Medicine, Odense University Hospital, Odense, Denmark; 2 Geriatric Research Unit, Department of Clinical Research, Odense University Hospital and University of Southern Denmark, Odense, Denmark; 3 Department of Geriatric Medicine, Odense University Hospital, Svendborg, Denmark; 4 DEFACTUM, Central Denmark Region, Aarhus, Denmark; 5 Department of Public Health, Aarhus University, Aarhus, Denmark; 6 Faculty of Health Sciences, University of Southern Denmark, Odense, Denmark; 7 Dutch WHO-Collaborating Centre, RIVM, Bilthoven, Netherlands

**Keywords:** activity and participation, capacity, ICF qualifiers, international classification of functioning, disability and health, performance, scoping review

## Abstract

**Background:**

The International Classification of Functioning, Disability and Health (ICF) provides a standardized rating system called “qualifiers” to describe capacity and performance, reflecting what a person can do and what a person does, respectively, yet their clinician-reported use remains conceptually and methodologically unclear.

**Objective:**

To map and describe clinician-reported assessments linked to capacity and performance qualifiers, and to examine how these qualifiers have been operationalized in practice.

**Methods:**

A scoping review was conducted following PRISMA-ScR guidelines. Systematic searches were performed in five bibliographic databases and grey literature sources from 2001 to August 2025. The protocol was registered prospectively on the Open Science Framework. Studies were included if they described clinician-reported assessments applying capacity and/or performance qualifiers in accordance with original ICF definitions.

**Results:**

Five studies met the inclusion criteria. Identified assessments were confined to a narrow range of Mobility categories, namely walking and use of the upper extremities. Most studies retrofitted existing clinical tests to qualifiers using heterogeneous approaches, including percentage-based thresholds, normative distributions, or clinically defined descriptors. Only one study developed an assessment specifically designed for qualifier use. Explicit differentiation between capacity and performance was rarely reported, and validation of qualifier thresholds was limited.

**Conclusions:**

Nearly a quarter of a century after the adoption of the ICF, clinician-reported use aligned with original ICF capacity and performance definitions remains limited, conceptually ambiguous, and methodologically inconsistent. Clearer conceptual definitions and internationally agreed, category-specific criteria are needed to enable meaningful, reliable, and comparable clinician-reported assessments of functioning.

## 1 Introduction

Human functioning is increasingly recognized as a central concept across multiple domains, including individual well-being, clinical practice, and health systems, and is increasingly proposed in the literature as a third key health indicator, alongside mortality and morbidity (1, 2).

The International Classification of Functioning, Disability and Health (ICF), has become the global standard for classifying functioning and disability (3). It provides a comprehensive, standardized classification and framework for understanding functioning and disability in a meaningful and interrelated way. The ICF serves as a scientific basis for understanding, describing, recording and studying health and health-related states, outcomes and determinants that permits comparison of data across countries and healthcare disciplines (4, 5).

The ICF is part of the World Health Organization's Family of International Classifications (WHO-FIC), which also includes the International Classification of Diseases (ICD) (6) and the International Classification of Health Interventions (ICHI) (7).

In the ICF, functioning is the umbrella term for body functions, body structures and activity and participation, while disability refers to impairments, limitations or restrictions in these components, referring to the negative aspects of the interaction between a person's health condition and that person's contextual factors (environmental and personal factors) which can be barriers or facilitators (4). In this paper, functioning is conceptualized in accordance with the ICF framework as a neutral and comprehensive construct, that includes not only positive aspects, but also disability-related aspects. For example, if the reference for walking without restriction is more than 250 meters, a person able to walk 100 meters, the parameter would indicate a severe restriction in walking. Thus, although the person has a restriction in walking, this still represents functioning, while at the same time representing disability.

Functioning is coded with an alphanumeric coding hierarchy. To be meaningful, an ICF code requires at least one qualifier (4). Qualifiers are ratings that indicate the extent, nature or location of a problem in functioning represented by a specific ICF code. The rating ranges from 0, indicating full functioning (no problem), to 4, indicating a complete problem, including the intermediate levels of mild, moderate and severe problems (4). Across all the components of functioning (body functions, body structures, activity and participation), the first qualifier indicates the extent of a problem in each functioning category, e.g the walking example above could be coded as d450.3 (severe restriction in walking).

In the activity and participation component, there are two standardized constructs; capacity and performance (3, 4). The performance construct describes what a person actually does in the person's current environment in light of the positive or negative impact of environmental factors (including all aspects of the physical, social and attitudinal environment) (4). The capacity construct, describes a person's inherent or intrinsic ability to execute a task or an action. This construct aims to indicate the highest probable level of functioning that a person may reach in a given domain at a given moment in a uniform or standard environment, and thus reflects the environmentally adjusted ability of the person. The two constructs are represented by four qualifiers (4):

- .x performance qualifier without environmental adjustment
- ._x capacity qualifier without assistive devices or personal assistance
- .__x capacity qualifier with assistive devices or personal assistance
- .___x performance qualifier without assistive devices or personal assistance

In the ICF book, it is not explicitly stated how qualifiers should be determined, apart from providing some qualifying terms and percentages. Regarding this, it is noted that “*For this quantification to be used in a universal manner, assessment procedures need to be developed through research*”. (4, p. 22).

In practice, the assessment of functioning remains complex. Despite the widespread use of instruments for assessing functioning, the assessment of functioning remains challenging (8), likely reflecting the inherent complexity of conceptualizing and monitoring functioning (9). Assessments of functioning should be grounded in the person's perspective, while recognizing that the most appropriate information source varies across ICF categories (4). In line with the ICF principle that coding should rely on explicit information (4), objective measurement is desirable where feasible, while subjective experience remains essential in many domains (5). To reflect this balance, we introduce the term *clinician-reported assessment* (ClinRA).

ClinRA is conceptualized as a structured health intervention, as it represents a professional action involving detection, analysis, and evaluation of functioning-related needs, consistent with the ICHI definition of assessment (7) and supported by WHO guidance in the ICF Practical Manual, which recognizes clinician observation and professional judgment as legitimate data sources (5). It denotes assessment procedures with operational criteria primarily based on professionally generated data derived from a single source or from a combination of sources, including tests, measurements, structured observations, and interviews, with selective integration of patient-reported information where appropriate (10, 11).

Here, more than two decades after the endorsement, the ICF has had great impact on the conceptual understanding of the complexity of functioning and stimulating new thinking, new applications in measurement and statistics (12, 13). The global application of the ICF aligns with its intended goals and principles, yet significant challenges have arisen, particularly regarding the use of ICF codes and qualifiers (12).

In this context, the need for standardized ClinRA criteria for rating capacity and performance qualifiers is particularly critical.

This scoping review aims to provide a comprehensive overview of ClinRAs linked to ICF capacity and performance qualifiers. Beyond mapping existing uses, the review critically examines and discusses the conceptual and methodological assumptions underpinning clinician-reported use of these qualifiers.

Review Questions

- In which categories within the activity and participation component of the ICF can ClinRAs applying the constructs of capacity and performance qualifiers be identified?
- Which of these ClinRAs have been specifically developed to evaluate functioning using the capacity and performance qualifiers?
- What theoretical concepts and frameworks informed the development and validation of these assessments?

## 2 Methods

### 2.1 Protocol and registration

This scoping review followed a protocol developed *a priori* and conducted in accordance with the Preferred Reporting Items for Systematic Reviews and Meta-Analyses extension for Scoping Reviews (PRISMA-ScR) guidelines (14). The protocol was registered on December 15, 2024, and is publicly accessible via the Open Science Framework (DOI: 10.17605/OSF.IO/93GSX).

### 2.2 Eligibility criteria

To be included, studies had to investigate or describe ClinRA linked to the ICF capacity and/or performance qualifiers and provide a scoring approach based on these qualifiers. In this review, the term studies is used as an umbrella term encompassing both peer-reviewed articles, clinical guidelines, and other types of reports.

Studies were excluded if they were not published in English. Studies were also excluded if they did not focus on ClinRA, for example, if they examined exclusively patient-reported outcomes, or if the use of capacity and performance qualifiers deviated from the original ICF definitions. Studies using the International Classification of Functioning, Disability and Health for Children and Youth (ICF-CY) or involving participants under the age of 18 were excluded, as the child and youth version is an adapted classification of the ICF that incorporates developmental considerations such as constructs for gaps and delays and age-specific categories, in line with a previous review (15).

### 2.3 Information sources

To identify potentially relevant literature, we systematically searched the following electronic bibliographic databases for records published from 2001 to August 2025: MEDLINE, Scopus, CINAHL, EMBASE, and PsycINFO.

To enhance the comprehensiveness of the review, we also conducted a grey literature search. We searched the websites of the World Health Organization (16) and the National Institute for Health and Care Excellence (17), screened the first 200 records retrieved via Google Scholar (18), consistent with recommendations by Haddaway et al. (19), and reviewed materials from the International Symposium on ICF Education (20), an initiative focused on the exchange and dissemination of ICF-related educational resources, to identify potentially relevant educational and implementation materials not indexed in traditional bibliographic databases.

All records identified through the search process in the bibliographic databases were imported into Covidence (Veritas Health Innovation, Melbourne, Australia) for screening and data management, and duplicates were removed automatically and manually when necessary. Studies identified via other methods were managed outside of Covidence and screened manually using the same eligibility criteria.

### 2.4 Search

The search strategy was developed with guidance from a research librarian at the University of Southern Denmark Library and reviewed by a health terminologist affiliated with a WHO-FIC Collaborating Centre (HtN). This process aimed to identify relevant keywords and subject headings and to ensure the methodological rigour of the search prior to implementation.

To enhance the identification of relevant studies, we conducted both forward and backward citation searches on all included articles, following established guidance on citation searching (TARCiS statement) (21). The literature search process was reported in accordance with the PRISMA for Searching guidelines (PRISMA-S) (22).

The Participants–Concept–Context criteria were defined as follows: adults (>18 years); ClinRA; use of ICF capacity and performance qualifiers. To maximize sensitivity, the search strategy did not apply restrictions related to the participant (age) or to the concept (ClinRA). A publication date filter from 2001 onwards was applied, corresponding to the introduction of the ICF.

The final search strategy is available in Supplementary File 1. Below is an example of the search conducted in Ovid MEDLINE(R):

1. ICF.mp. or exp “International Classification of Functioning, Disability and Health”/
2. International Classification of Functioning.mp.
3. (International Classification of Functioning, Disability and Health).mp. [mp = title, book title, abstract, original title, name of substance word, subject heading word, floating sub-heading word, keyword heading word, organism supplementary concept word, protocol supplementary concept word, rare disease supplementary concept word, unique identifier, synonyms, population supplementary concept word, anatomy supplementary concept word]
4. 1 or 2 or 3
5. Qualifier*.mp.
6. Capacity.mp.
7. Performance.mp.
8. 5 or 6 or 7
9. 4 and 8
10. limit 9 to yr = “2001 -Current”

### 2.5 Selection of sources of evidence

All titles and abstracts from bibliographic databases were independently screened by two reviewers: reviewer 1 (TK) and reviewer 2 (performed by JGP, TM, or KH). Studies identified via other methods were screened only by reviewer 1 (TK). To address conflicts between reviewers during the title–abstract screening, a random sample of approximately 10% of records was used to identify and thematically analyze discrepancies in the review process. Based on this analysis one reviewer (TK) presented categories of conflicts for discussion at regular review team meetings. These discussions informed a set of resolution principles, which was subsequently applied to consistently resolve all identified conflicts. In cases where conflicts were not represented in the thematic analysis, they were included for full-text reading.

Full-text articles were subsequently assessed for eligibility by reviewer 1 (TK), who documented the reason for exclusion for each excluded article. These reasons were verified by reviewer 2 (JGP, TM, or KH) to ensure transparency and accuracy. In cases of uncertainty, eligibility decisions were discussed collaboratively within the review team until consensus was reached.

The entire process was supervised by two ICF experts (HtN and TM). A third reviewer (LRM) could be consulted in case of unresolved disagreements.

### 2.6 Data charting process

A data extraction form was developed in Microsoft Excel 2016 (Microsoft Corporation, Redmond, WA; 32-bit) based on the items descripted in the protocol, pilot tested, and revised as needed to systematically organize the information retrieved from each study included. The data extraction was done by one reviewer (TK), and was verified by a second reviewer (JGP).

### 2.7 Data items

We extracted data on article characteristics, including author(s), publication year, title, DOI, publisher, and references used for backward citation searches. We recorded methodological and contextual variables on study design, materials/setting/population, tools required and the name and type of clinician-reported assessment intervention. We registered conceptual variables including construct, the related ICF activity/participation category, the stated purpose, and ICF qualifier design intent (i.e., whether the ClinRA procedure was designed for, or adapted to, rating capacity and performance qualifiers). Finally, we captured analytic and reporting elements: linking/coding method, validation method, reported strengths, reported limitations, and key findings.

### 2.8 Synthesis of results

All relevant charted data were synthesized descriptively and are presented narratively and in tabular form (Tables 1, 2). Table 1 provides an overview of the ICF categories and corresponding ClinRAs, specifying the type of assessment, required tools or equipment, the underlying construct (capacity or performance), and whether the ClinRA was designed for, or adapted to, the use of ICF qualifiers. Table 2 outlines study-specific methods for operationalizing results into qualifier levels, including the coding approach, operational criteria, population characteristics, and reported validation procedures.

**Table 1:** ICF categories and corresponding clinician-reported assessments.

ICF category	Clinician-reported assessment tool [type of intervention] (Study)	Tools/equipment required	Construct	Designed for/adapted to ICF qualifiers
Second-level categories				
d430 Lifting and carrying objects	*Not named* [observation] Grill et al. (27)	*Not reported*	*Not reported*	Designed for qualifiers
d440 Fine hand use	Minnesota Dexterity Test [Test] Caporaso et al. (26)	Minnesota Dexterity Test kit, three depth-sensor cameras, desk and chair.	*Not reported* (capacity implicit^c^)	Adapted to qualifiers
d450 Walking	The Six-Minute Walk Test [Test] Dalavina et al., (24)	30-m corridor, cones, stopwatch, standardized instructions.	*Not reported* (capacity implicit^c^)	Adapted to qualifiers
Third-level categories				
d4500 Walking short distances	10-meter Walk Test [Test] Benito García et al. (25)	10-m corridor, floor markings, stopwatch.^a^	*Not reported*	Adapted to qualifiers
	Incremental shuttle walk test [Test] Peres Costa et al. (23)	10-m corridor, cones, standardized audio signal (beep test), stopwatch.	*Not reported* (capacity implicit^c^)	Adapted to qualifiers
d4501 Walking long distances	The Six-Minute Walk Test [Test] Benito García et al. (25)	30-m corridor, cones, stopwatch, standardized instructions.^a^	*Not reported* (capacity implicit^c^)	Adapted to qualifiers
d4502 Walking on different surfaces	Modified Emory Functional Ambulation Profile: part carpet [Test] Benito García et al. (25)	5-meter walk on a carpeted surface, marked start/finish lines, stopwatch.^a^	*Not reported* (performance implicit^b^)	Adapted to qualifiers
d4503 Walking around obstacles	Modified Emory Functional Ambulation Profile: part obstacles [Test] Benito García et al. (25)	Standardized obstacle course, marked start/finish lines, stopwatch.^a^	*Not reported* (performance implicit^b^)	Adapted to qualifiers

a Not reported in article; identified from external sources.

b Performance implicit: normal, comfortable speed.

c Capacity implicit: the highest probable level of functioning that a person may reach in a standard environment.

**Table 2:** Methods for operationalizing ICF capacity and performance qualifiers.

Study	Linking/coding method	Explicit criteria for ICF qualifiers	Population	Validation method
Peres Costa et al. (23)	ICF qualifiers were operationalized by recoding the percentage of predicted ISWT distance into qualifiers.	.0 = 96%–100% of predicted ISWT .1 = 76%–95% of predicted ISWT .2 = 51%–75% of predicted ISWT .3 = 5%–50% of predicted ISWT .4 = 0%–4% of predicted ISWT	57 adults aged 20–70 years (mean 46.15, SD 14.12; 40 female) with difficult-to-treat asthma, clinically stable ≥3 months and receiving outpatient treatment; recruited from an ambulatory center in São Paulo, Brazil.	No validation of the qualifiers
Benito García et al. (25)	ICF qualifiers were operationalized by converting the test score into percentages of normative reference values.	.0 = 0%–4% of the normative reference values .1 = 5%–24% of the normative reference values .2 = 25%–49% of the normative reference values .3 = 50%–95% of the normative reference values .4 = 96%–100% of the normative reference values	24 adults aged >18 years (mean 65.6, SD 10.73; 10 females) 1.5–5 years post-stroke; recruited from an outpatient neurorehabilitation centre in Madrid, Spain.	No validation of the qualifiers
Grill et al. (27)	ICF qualifiers were defined based on clinically meaningful definitions of the patient's ability to lift and carry objects, with each mapped to a specific qualifier.	.0 = patient is able to lift and carry heavy objects .1 = patient is able to lift a heavy object .2 = patient is able to lift and carry a light object (e.g., a bottle) .3 = patient is able to lift a light object .4 = patient is not able to lift or to carry.	25 adults requiring physical therapy for neurological, musculoskeletal or cardiopulmonary conditions; recruited from an acute hospital in Zurich, Switzerland (demographic data not reported).	Inter-observer reliability analysis using raw agreement, specific agreement, kappa and weighted kappa statistics, Bangdiwala agreement chart, and hierarchical log-linear modelling of ordinal agreement.
Caporaso et al. (26)	ICF qualifiers were operationalized by categorizing task duration into intervals based on standard deviations from the mean.	.0 ≤ 99.09 s (<1 SD) .1 = 99.10 s to 108.81 s (1 SD to 2 SD) .2 = 108.82 s to 118.53 s (2 SD to 3 SD) .3 = 118.54 s to 128.25 s (3 SD to 4 SD) .4 ≥ 128.26 s (>4 SD)	10 young healthy adults (mean age 24 ± 4 years); recruited for experimental testing at the ErgoS Lab, University of Naples Federico II, Italy.	No validation of the qualifiers (*Technical/algorithmic validation with manual visual assessment as reference*)
Dalavina et al. (24)	ICF qualifiers were operationalized by recoding the percentage of predicted 6MWT distance into qualifier.	.0 = 96%–100% of predicted 6 MWT .1 = 76%–95% of predicted 6 MWT .2 = 51%–75% of predicted 6 MWT .3 = 5%–50% of predicted 6 MWT .4 = 0%–4% of predicted 6 MWT	33 adults aged 20–70 years (mean 39.4, SD 17.3; 24 females) with pulmonary hypertension, clinically stable ≥3 months and receiving outpatient medical treatment; recruited consecutively from the Cardiopulmonary Rehabilitation Laboratory at UNINOVE in São Paulo, Brazil.	No validation of the qualifiers (A*ssociation analysis between the new ICF-based classification and the WHO Functional Class*)

SD, standard deviations; s, seconds; predicted 6 MWT: predicted distance in the Six-Minute Walk Test with the equations proposed by Dourado (55) DPmTC6pred=299.296−(2.728xage)−(2.160xweight)+(361.731xheight)+(56.386xsex)),wheremale=1,female=0; predicted ISWT: Incremental shuttle walk test with the equations proposed by Probst et al. (56) ISWTpred=1449.701−(11.735xage)+(241.897xgender)−(5.686xBMI),wheremale=1,female=0.

### 2.9 Changes from the registered protocol

Following publication of the protocol, two methodological amendments were introduced. First, an additional exclusion criterion was applied whereby studies were excluded if the application of capacity and performance qualifiers deviated from the original ICF definitions. This was done to ensure conceptual consistency and avoid conflation of distinct logics. Studies were therefore excluded if qualifiers were applied using modified scales or graded according to the level of assistance required.

Second, an additional inclusion requirement was introduced, requiring studies to describe their scoring approach in sufficient detail to show how qualifiers were operationalized, enable meaningful comparison and synthesis.

## 3 Results

**Figure 1:**
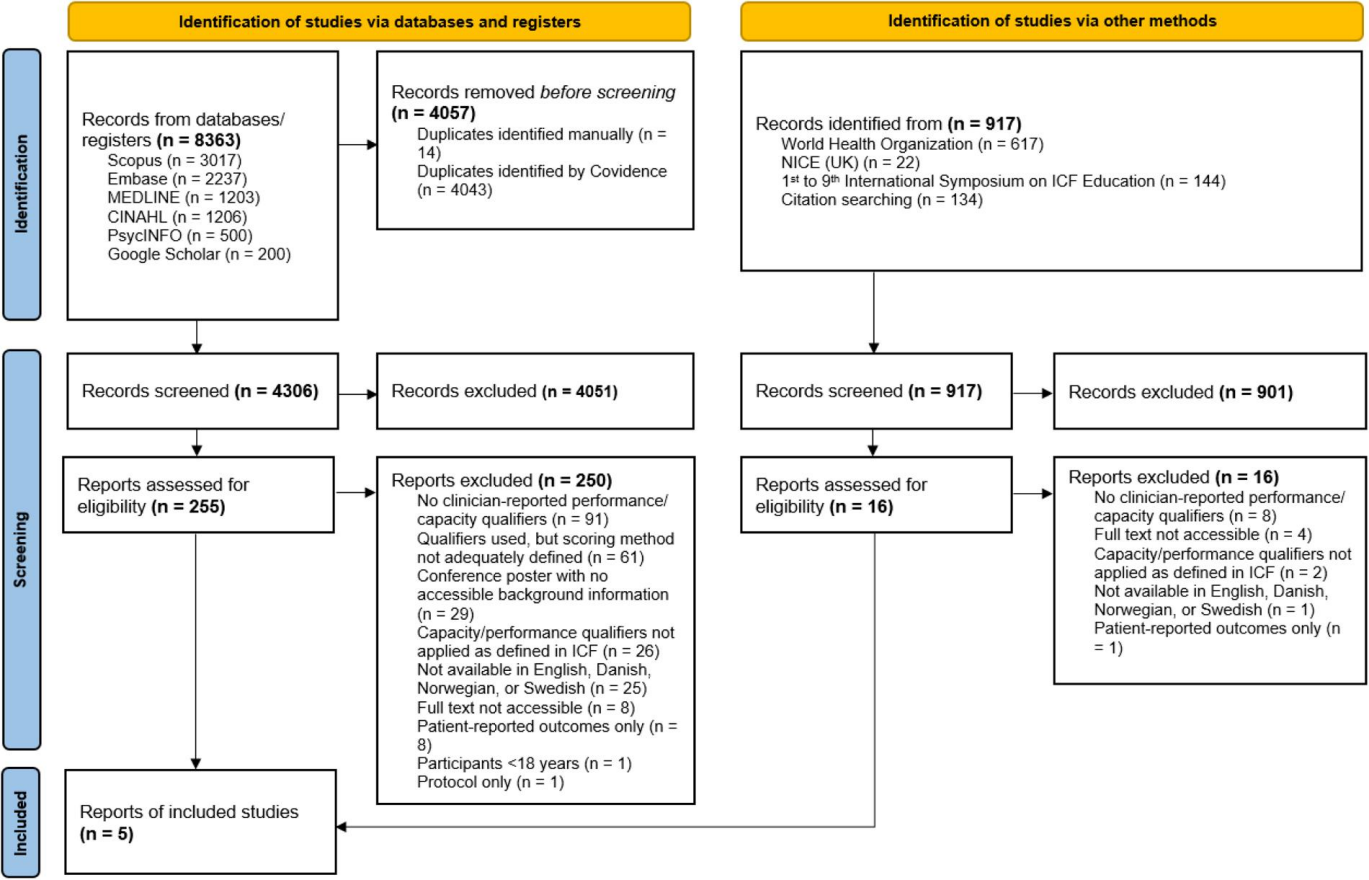
PRISMA flow diagram.

The literature search yielded a total of 9,280 records. After removal of duplicates, 5,223 records were screened by title and abstract. Of these, 271 records were retrieved for full-text assessment. Following full-text screening, 266 articles were excluded for not meeting the eligibility criteria. In total, five studies met the inclusion criteria. The study selection process and reasons for exclusion at the full-text stage are summarized in the PRISMA flow diagram (Figure 1).

### 3.1 Results synthesis

#### 3.1.1 Activity and participation categories with clinician-reported assessments applying capacity and performance qualifiers

Across the five included studies, ClinRAs applying ICF qualifiers were identified only within a limited set of categories in the Mobility chapter of the activity and participation component. These included three second-level categories; d430 Lifting and carrying objects, d440 Fine hand use, and d450 Walking, and four third-level categories; d4500 Walking short distances, d4501 Walking long distances, d4502 Walking on different surfaces, and d4503 Walking around obstacles.

##### 3.1.1.1. Clinician-reported assessment tools and underlying constructs

Among the five included studies, four relied on pre-existing ClinRA instruments. These comprised standardized walking assessments, the Incremental Shuttle Walk Test (23), the Six-Minute Walk Test (24, 25), and the Modified Emory Functional Ambulation Profile and 10-meter Walk Test (25), as well as the Minnesota Dexterity Test for fine hand use (26). Grill et al. (27) differed by developing a new observation-based ClinRA.

Although Benito García et al. (25) explicitly stated that the assessment reflected “real performance”, it was not reported whether the 10-Meter Walk Test was intended to represent fast or comfortable walking speed. Apart from this exception, none of the included studies explicitly stated whether the assigned qualifiers reflected capacity or performance, nor whether assessments were conducted with or without assistive devices or personal assistance.

Across the included studies, the interpretation of whether the ClinRAs reflected capacity or performance was inferred from the nature of the applied test conditions. The Six-Minute Walk Test, the Incremental Shuttle Walk Test, and the Minnesota Dexterity Test were interpreted as implicitly assessing capacity, as these tests aim to capture the person's highest probable level of functioning under standardized conditions. The Modified Emory Functional Ambulation Profile subtests involving carpet and obstacles were interpreted as implicitly reflecting performance, as these assessments involve walking at a normal, comfortable speed rather than the highest probable level of functioning. In contrast, the 10-Meter Walk Test and the unnamed observation-based assessment could not be conclusively classified as either capacity or performance, as the studies did not explicitly state whether the assessments were intended to represent the person's highest probable level of functioning.

#### 3.1.2 Methodological approaches for developing clinician-reported assessments to rate capacity and performance qualifiers

Across the included studies, two distinct approaches to applying ICF qualifiers were identified. Grill et al. (27) used clinically meaningful descriptions designed to apply ICF qualifiers. All remaining studies (23–26) retrofitted existing tests by converting test scores into ICF qualifiers (see Section 3.1.3.1 for examples).

#### 3.1.3 Theoretical concepts and frameworks informing the development and validation of capacity and performance qualifier rating criteria

##### 3.1.3.1 Methods for linking and coding test results to capacity and performance qualifiers

Across studies, heterogeneous approaches were used to operationalize ICF qualifiers. Test results were linked to qualifiers using three main strategies: percentage-based thresholds derived from predicted or normative reference values, clinically defined descriptor categories, and distribution-based statistical intervals.

###### 3.1.3.1.1 Percentage-based thresholds

Three studies operationalized ICF qualifiers by transforming test outcomes into percentage-based categories relative to predicted or normative reference values. Peres Costa et al. used the Incremental Shuttle Walk Test and Dalavina et al. used the Six-Minute Walk Test, with both studies applying predefined percentage ranges of predicted values derived from population-specific equations (23, 24). Benito García et al. used the Six-Minute Walk Test together with the Modified Emory Functional Ambulation Profile and the 10-Meter Walk Test, deriving qualifiers from percentages of established normative reference values (25).

###### 3.1.3.1.2 Clinically defined descriptors

One study, conducted by Grill et al., operationalized ICF qualifiers using predefined clinical descriptions. Qualifier levels were specified through descriptions of the person's ability to lift and carry objects of different weights, with each description directly mapped to a corresponding ICF qualifier category (27).

###### 3.1.3.1.3 Distribution-based statistical intervals

One study, conducted by Caporaso et al. operationalized ICF qualifiers by categorizing test outcomes into predefined time intervals corresponding to increasing standard deviation ranges in a reference sample. Total task duration on the Minnesota Dexterity Test was classified into successive qualifier levels spanning one to more than four standard deviations (26).

##### 3.1.3.2 Assessment tools and equipment

Across the included studies, the majority of ClinRAs relied on low-technology, clinically accessible equipment. Most walking assessments were conducted in flat, short corridors (≤30 m) using simple spatial markers (e.g., floor markings or cones) and a stopwatch. Externally paced auditory cues were applied as part of the Incremental Shuttle Walk Test (23). Assessment of walking across different surfaces and around obstacles was based on predefined obstacle courses and carpeted walkways (25). Advanced instrumentation was employed in one study conducted by Caporaso et al., combining the Minnesota Dexterity Test with a multi-depth sensor camera system for automated motion capture (26). In one study conducted by Grill et al., no specific equipment was reported (27).

##### 3.1.3.3 Validation approaches

Validation of ICF-based qualifiers was generally limited and heterogeneous. Several studies applied qualifiers without conducting any formal validation of the qualifier thresholds or scale properties themselves (23–25). In one study, Dalavina et al. explored associations between classification using the ICF and the WHO Functional Class (WHO-FC), a four-level classification of symptom severity used in pulmonary hypertension, however, this was not presented as a systematic validation of the qualifier scale (24). In one study, Caporaso et al. evaluated an automated dexterity assessment by comparing algorithm-generated classifications with clinicians' visual assessments, thereby addressing technical performance rather than construct validity of the qualifiers (26).

The most extensive psychometric evaluation was reported by Grill et al., focused on inter-observer agreement and ordinal scale properties. This work examined inter-rater reliability using multiple agreement statistics, including raw agreement, specific agreement, kappa and weighted kappa coefficients, agreement charts, and hierarchical log-linear models (27). However, even in this case, the analyses primarily addressed the consistency of rater use of qualifiers rather than validating the qualifiers as indicators of underlying capacity or performance constructs.

#### 3.1.4 Key findings from included studies

The key findings reported in each included study addressed different applications of ICF qualifiers to ClinRAs.

Peres Costa et al. reported that ICF qualifiers were useful for categorizing scores from the Incremental Shuttle Walk Test by enabling a more structured representation of functioning in relation to predicted values. The authors reported that this approach supported evaluation, follow-up, and clinical decision-making in rehabilitation and highlighted its potential to strengthen the integration and use of ICF concepts in clinical practice, as well as in research and health services (23).

Benito García et al. reported that ICF qualifiers were useful for capturing functioning information and were sensitive to detecting post-intervention changes in walking-related activities in persons with chronic stroke. The authors also reported conceptual limitations, noting that the clinical tests used to derive ICF qualifiers did not fully correspond to the conceptual definitions of the linked ICF categories (e.g., the Six-Minute Walk Test does not represent “walking long distances”, defined in the ICF as distances greater than 1 km). Notably, although walking short distances improved in raw test scores, this improvement did not reach statistical significance at the level of the ICF qualifiers (25).

Grill et al. reported limited to moderate interobserver agreement when applying ICF qualifiers. Raw agreement was 0.52, and chance-corrected agreement (kappa) was 0.36, indicating fair agreement. Weighted kappa coefficients ranged from 0.51 (Cicchetti & Allison weights) to 0.63 (Fleiss & Cohen weights), reflecting moderate agreement. Agreement was highest for qualifier categories .0, .1, and .4, whereas observers had difficulties differentiating between qualifier categories .2 and .3. Log-linear modeling showed that the strength of agreement was not homogeneous across qualifier categories, suggesting that the operationalization of the qualifier scale influenced reliability. The authors concluded that low agreement should prompt further investigation of underlying reasons and that cross-tabulations, graphical methods, and modeling may provide valuable insights to improve the scale under examination (27).

Caporaso et al. demonstrated that a proposed automated tool for the Minnesota Dexterity Test was able to automatically assess dexterity performance using depth-camera data. According to the authors, the tool showed potential to assist clinicians by providing automated outcome measures, although no further claims regarding clinical application were made (26).

Finally, Dalavina et al. showed that applying ICF qualifiers to Six-Minute Walk Test scores provided a clinically meaningful, individualized alternative to the WHO-FC in pulmonary hypertension. The authors reported that by contrasting expected and actual walking distance, the ICF-based approach captured limitations that may not be reflected in WHO-FC categories. Although no statistical association was found between the two classification approaches, the ICF qualifiers was reported to offer added clinical nuance and finer differentiation of limitation, supporting more precise monitoring, clinical decision-making, and targeted rehabilitation planning (24).

## 4 Discussion

### 4.1 Summary of evidence

In this scoping review, we screened more than 5,000 records and identified only five studies applying eight ClinRAs to ICF capacity or performance qualifiers consistent with WHO definitions. All identified uses were concentrated within a narrow set of categories in the Mobility chapter of the activity and participation component. No studies were found for any categories outside mobility, indicating that the practical use of these qualifiers in ClinRA remains extremely limited in scope.

### 4.2 Debates and challenges in the use of ICF qualifiers

The use of ICF qualifiers has long been subject to debate and criticism (28). Previous studies have shown low to moderate inter-rater and intra-rater reliability when qualifiers are applied without modification (29–32) and have shown that clinicians experience considerable difficulties in implementation due to the lack of clear operational guidelines (33), particularly when applying qualifiers at the level of individual categories within the activity and participation component (34). At the same time, qualifiers are considered central to the operationalization of the ICF (15) and are expected to strengthen its clinical applicability, although concerns regarding reliability and validity persist (35). Consequently, several authors have emphasized the need for further research on clearer definitions and validation of the qualifiers (8, 36–42).

Findings from this review reinforce these concerns regarding conceptual issues and methodological challenges related to the definition and operationalization of ICF qualifiers.

#### 4.2.1 The conceptual issue with the constructs capacity and performance

Across the five included studies, capacity and performance were rarely defined explicitly, and construct classification relied mainly on implicit assumptions inferred from test conditions. This reflects previously described conceptual ambiguities (43, 44). For example, although one study (25) explicitly stated that the assessment reflected “real performance”, it remained unclear whether the 10-Meter Walk Test was intended to represent fast or comfortable walking speed. Apart from this exception, none of the included studies explicitly reported whether the assigned qualifiers reflected capacity or performance, nor whether assessments were conducted with or without assistive devices or personal assistance. This lack of explicit reporting compromises the interpretability and comparability of the applied qualifiers.

Beyond the studies included in this review, similar ambiguities were also evident in the large number of records excluded due to applications of qualifiers that were inconsistent with ICF definitions of capacity and performance. This included the use of instruments such as the Mini-ICF, Functional Independence Measure, and Barthel Index, which do not directly operationalize these constructs as defined by the ICF. In the absence of clear operational guidance, clinicians and researchers appear to develop *ad hoc* solutions that may be locally useful but risk undermining the intended use of the qualifiers for category within the activity and participation component. This problem may be reinforced by ambiguities within the ICF book itself, including partly overlapping definitions of capacity and performance and the use of additional qualifiers (4), which may contribute to inconsistent interpretation and application in clinical practice. In literature, the distinction between capacity and performance is often interpreted primarily through the impact of environmental factors (45–47). However, in our interpretation, this perspective does not fully align with the qualifier structure in the ICF, since the constructs of capacity and performance may be operationalized both with and without the use of assistive devices or personal assistance. Consequently, the conceptual distinction between the constructs may not be reducible solely to environmental influence.

Clearer operationalization is therefore advisable if the qualifiers are to function as meaningful classification constructs. One possible approach is to conceptualize capacity and performance as non-overlapping and jointly exhaustive, consistent with general principles of classification aimed at reducing conceptual ambiguity and the risk of misclassification (48). One possible interpretation could be to understand capacity strictly as “the highest probable level of functioning that a person may reach in a standard environment”, whereas performance could encompass all functioning other than that. Importantly, both constructs would be defined independently of the use of assistive devices or personal assistance. Such an operationalization would reduce conceptual overlap, align more closely with the ICF's stated intentions, and ensure that each assessment can be classified unambiguously as reflecting one perspective or the other.

However, full operationalization of ICF may also prompt consideration of establishing additional standardized constructs, such as those commonly used for capturing patient-reported outcomes or for grading the extent and nature of personal assistance. Yet a construct for grading the extent and nature of personal assistance is not necessarily advisable, as it risks introducing inconsistencies with the ICF's classification of environmental factors. Nevertheless, reflecting on these possibilities may help clarify conceptual boundaries and support a more coherent and operationally consistent use of the qualifiers in clinical practice and research.

Another challenge for the constructs of capacity and performance is their overlap with other established terminology. In clinical practice, these perspectives are often blurred; for example, a test may be labelled a “performance-based measure” without reflecting the ICF construct of performance. This conceptual confusion between performance and performance-based measurement has also been noted in the literature (49). Similar ambiguity was evident in our review (25).

Such inconsistencies risk obscuring the conceptual distinctions that qualifiers are intended to capture, thereby complicating their meaningful application. A coherent, shared interpretation of capacity and performance is therefore a prerequisite for future attempts to standardize ClinRA procedures.

#### 4.2.2 Methodological challenges in operationalizing qualifiers

Beyond the conceptual problems, this review reveals methodological heterogeneity in the operationalization of ICF qualifiers across ClinRAs. Qualifiers were derived using diverse approaches, including percentages of predicted values, normative reference distributions, standard deviation bands, and clinically defined descriptors. Importantly, the observed methodological diversity does not in itself imply conceptual inconsistency, as the applied approaches are broadly aligned with the definition in the ICF book of limitation or restriction as the discordance between observed and expected functioning (4). While this variation may reflect both the complexity of the construct and the lack of consensus on qualifier operationalization, it nevertheless can compromise the comparability, interpretability, and scientific credibility of the qualifiers.

Notably none of the included studies validated whether assigned qualifiers corresponded to individuals' self-perception or to any external reference standard. Validation efforts largely focused on rater agreement or technical accuracy rather than construct validity or the clinical relevance and qualifier thresholds, with one exception which showed higher agreement for qualifier categories .0, .1, and .4, and persistent difficulties differentiating between .2 and .3, consistent with previous studies (50).

Collectively, these findings suggest that current applications risk reducing qualifiers to technically convenient but conceptually weak labels, underscoring the need for clearer conceptual delineation and category-specific operational criteria.

### 4.3 Perspectives on the complexity of human functioning and its implications for standardized assessment

The lack of standardization in how qualifiers are applied in practice reflects a broader international issue, as highlighted by the National Academy of Medicine commentary “Standardizing Assessment and Reporting of Functioning Information for Rehabilitation and Healthy Aging” (37). The report emphasizes that advancing the “functioning revolution” requires a shared language and a common metrics across professions, sectors, and countries, supported by professional consensus, specialized competence, and clear methodological guidance.

Accordingly, the establishment of a more consistent approach to ClinRA of functioning may require the development of category-specific manuals for the use of ICF qualifiers (8). Ideally, validated assessment criteria should be established for each relevant category within the activity and participation component, comparable to the diagnostic criteria developed for certain ICD diagnoses and disseminated through clinical guidelines, manuals, and peer-reviewed publications (51). Such manuals could operationalize ICF concepts at a level that preserves theoretical rigor while ensuring clinical applicability. To guarantee quality and professional anchoring, this should be regarded as a specialist task requiring advanced knowledge of both classification principles and assessment practices (52).

A common feature of the included studies in this review is that functioning within a given ICF category is typically evaluated based on a single parameter, derived from one clinician-reported measurement instrument. While such approaches may enhance feasibility and reproducibility, they raise the question of whether this represents an overly reductionist understanding of human functioning. Humans, like other organisms, live, maintain, and adapt within ecological niches, and their functioning is shaped by health conditions (e.g., diseases, disorders, injuries, and traumas) as well as contextual factors. Therefore, human functioning is widely recognized as a complex phenomenon that both affects and is affected by multiple interacting factors (53, 54).

From this perspective, the assessment of functioning within a specific ICF category may be more appropriately conceptualized as an integrative synthesis of multiple sources of information rather than as a single measurement. Such assessments combine e.g., tests, measurements, observations, and interviews with data from patient-reported questionnaires, clinical examinations, technical investigations, and relevant case histories (10).

For example, an assessment of functioning within an ICF walking category may involve an integrated evaluation of spatiotemporal parameters such as gait speed, step length, and cadence; gait-related phenomena, such as visuospatial disorientation, instability, and compensatory movement patterns; and subjective elements, encompassing sensations, confidence, and cognitive aspects related to walking. Together, these dimensions may provide essential information for interpreting functioning within the specific ICF category.

Such multidimensional considerations illustrate that assigning an ICF qualifier is not merely a technical act but a complex, professionally grounded interpretive process. This process should be carried out on the basis of professional judgment supported by internationally agreed, category-specific manuals, which clarify relevant sources of information and how these should be systematically integrated into the assessment.

A structured approach of this kind may enhance comparability and data quality and could represent a concrete step toward the functioning revolution called for in the Lucerne report, in which functioning becomes an explicit and systematic health indicator on par with disease and mortality (37).

### 4.4 Limitations and strengths of the review

A key limitation of this review is the introduction of the umbrella concept ClinRA. Although anchored in the ICHI definition of assessment (7) and informed by the ICF Core Set—Manual for Clinical Practice (10), ClinRA remains a novel and unvalidated term in rehabilitation research. While introduced to distinguish clinician-derived assessments from purely patient-reported measures, its deliberately broad scope—encompassing tests, measurements, structured observations, and interviews—may overlap with existing assessment constructs and limit comparability across studies. ClinRA should therefore be regarded as a preliminary conceptual proposal, warranting critical appraisal and further theoretical and empirical development.

Despite a comprehensive multi-database search, the resulting evidence base was limited. This likely reflects a sparsely investigated field but may also be attributable to variability in terminology and inconsistent use of concepts. This could potentially also be explained by the fact that studies identified through methods other than bibliographic database searches were screened by only one reviewer. Consequently, relevant studies may have been missed.

The exclusion of ICF-CY and populations <18 years may have resulted in a substantial risk of omitting relevant insights into how capacity and performance qualifiers have been operationalized and developed in ClinRAs.

Finally, the strict inclusion criteria for ICF capacity and performance qualifiers, excluding modified grading logics (e.g., assistance-based scales), ensured conceptual clarity but may have resulted in the exclusion of clinically meaningful adaptations. As a result, alternative applications of qualifiers are not represented in this review.

A key strength of this scoping review is its strict conceptual adherence to the original definitions of ICF constructs capacity and performance, which ensured internal coherence and conceptual clarity in the synthesis. The review was conducted according to a registered protocol and followed PRISMA-ScR guidance, using a comprehensive search strategy across multiple databases and grey literature sources, thereby enhancing transparency and methodological rigor. Moreover, to our knowledge, this is the first review to specifically examine ClinRA directly linked to ICF capacity and performance qualifiers, addressing an important and underexplored gap in the literature.

## 5 Conclusions

Nearly a quarter of a century after the adoption of the ICF, research applying clinician-reported assessments to inform the rating of capacity and performance qualifiers remains limited. In this review, eligible evidence was confined to a small number of studies focusing exclusively on mobility-related categories, and characterized by substantial heterogeneity in the operationalization of qualifiers and minimal validation of the underlying constructs. Capacity and performance were rarely defined explicitly, and qualifier assignment was largely based on implicit assumptions derived from test conditions.

Together, these findings indicate that the methodological foundations required to support meaningful and standardized clinician-reported assessments at the category level are still insufficient. Addressing this gap will require clearer conceptual definitions and broader international consensus on validated, category-specific operational criteria. Such developments are necessary to enable the generation of robust and comparable data on functioning and disability for both research and clinical practice.
