# Supplementary material for: Exploring clinician-reported assessments of capacity and performance qualifiers in the ICF: a scoping review
Source: Front Rehabil Sci. 2026 May 7;7:1792865. doi: 10.3389/fresc.2026.1792865 (PMC13191736; doi:10.3389/fresc.2026.1792865)
Supplement: Supplementary file 1 [file Supplementaryfile1.docx]

Supplementary File 1: Search strategy

# Ovid MEDLINE(R) ALL <1946 to December 13, 2024> (Literature Search performed: December 16, 2024)

| **Search name** | **Search query** | **Results** |
| --- | --- | --- |
| 1 | ICF.mp. or exp "International Classification of Functioning, Disability and Health"/ | 6279 |
| 2 | International Classification of Functioning.mp. | 4431 |
| 3 | (International Classification of Functioning, Disability and Health).mp. [mp=title, book title, abstract, original title, name of substance word, subject heading word, floating sub-heading word, keyword heading word, organism supplementary concept word, protocol supplementary concept word, rare disease supplementary concept word, unique identifier, synonyms, population supplementary concept word, anatomy supplementary concept word] | 3990 |
| 4 | 1 or 2 or 3 | 7377 |
| 5 | Qualifier*.mp. | 908 |
| 6 | Capacity.mp. | 755593 |
| 7 | Performance.mp. | 1555124 |
| 8 | 5 or 6 or 7 | 2226337 |
| 9 | 4 and 8 | 1074 |
| 10 | limit 9 to yr="2001 -Current" | 1061 |

## Supplementary literature search performed August 21, 2025 (Ovid MEDLINE, limited to December 2024 – current).

| **Search name** | **Search query** | **Results** |
| --- | --- | --- |
| 1 | ICF.mp. or exp "International Classification of Functioning, Disability and Health"/ | 6555 |
| 2 | International Classification of Functioning.mp. | 4636 |
| 3 | (International Classification of Functioning, Disability and Health).mp. [mp=title, book title, abstract, original title, name of substance word, subject heading word, floating sub-heading word, keyword heading word, organism supplementary concept word, protocol supplementary concept word, rare disease supplementary concept word, unique identifier, synonyms, population supplementary concept word, anatomy supplementary concept word] | 4165 |
| 4 | 1 or 2 or 3 | 7710 |
| 5 | Qualifier*.mp. | 933 |
| 6 | Capacity.mp. | 793633 |
| 7 | Performance.mp. | 1667777 |
| 8 | 5 or 6 or 7 | 2368903 |
| 9 | 4 and 8 | 1129 |
| 10 | limit 9 to yr="2024 -Current" | 142 |

# Embase Classic+Embase <1947 to 2024 December 13> (Literature Search performed: December 16, 2024)

| **Search name** | **Search query** | **Results** |
| --- | --- | --- |
| 1 | ICF.mp. or exp "International Classification of Functioning, Disability and Health"/ | 9970 |
| 2 | International Classification of Functioning.mp. | 6425 |
| 3 | (International Classification of Functioning, Disability and Health).mp. [mp=title, abstract, heading word, drug trade name, original title, device manufacturer, drug manufacturer, device trade name, keyword heading word, floating subheading word, candidate term word] | 5771 |
| 4 | 1 or 2 or 3 | 10853 |
| 5 | Qualifier*.mp. | 1233 |
| 6 | Capacity.mp. | 1038190 |
| 7 | Performance.mp. | 2307753 |
| 8 | 5 or 6 or 7 | 3227643 |
| 9 | 4 and 8 | 2020 |
| 10 | limit 9 to yr="2001 -Current" | 1999 |

## Supplementary literature search performed August 21, 2025 (Embase Classic+Embase, limited to 2024 – current).

| **Search name** | **Search query** | **Results** |
| --- | --- | --- |
| 1 | ICF.mp. or exp "International Classification of Functioning, Disability and Health"/ | 11378 |
| 2 | International Classification of Functioning.mp. | 6836 |
| 3 | (International Classification of Functioning, Disability and Health).mp. [mp=title, abstract, heading word, drug trade name, original title, device manufacturer, drug manufacturer, device trade name, keyword heading word, floating subheading word, candidate term word] | 6123 |
| 4 | 1 or 2 or 3 | 12304 |
| 5 | Qualifier*.mp. | 1284 |
| 6 | Capacity.mp. | 1103922 |
| 7 | Performance.mp. | 2492842 |
| 8 | 5 or 6 or 7 | 3465159 |
| 9 | 4 and 8 | 2384 |
| 10 | limit 9 to yr="2024 -Current" | 238 |

# APA PsycInfo <1806 to December 2024 Week 2> (Literature Search performed: December 16, 2024)

| **Search name** | **Search query** | **Results** |
| --- | --- | --- |
| 1 | ICF.mp. | 2281 |
| 2 | International Classification of Functioning.mp. | 1998 |
| 3 | (International Classification of Functioning, Disability and Health).mp. [mp=title, abstract, heading word, table of contents, key concepts, original title, tests & measures, mesh word] | 1767 |
| 4 | 1 or 2 or 3 | 2853 |
| 5 | Qualifier*.mp. | 488 |
| 6 | Capacity.mp. | 109443 |
| 7 | Performance.mp. | 505087 |
| 8 | 5 or 6 or 7 | 597406 |
| 9 | 4 and 8 | 475 |
| 10 | limit 9 to yr="2001 -Current" | 470 |

## Supplementary literature search performed August 21, 2025 (APA PsycInfo, limited to 2024 – current).

| **Search name** | **Search query** | **Results** |
| --- | --- | --- |
| 1 | ICF.mp. | 2355 |
| 2 | International Classification of Functioning.mp. | 2068 |
| 3 | (International Classification of Functioning, Disability and Health).mp. [mp=title, abstract, heading word, table of contents, key concepts, original title, tests & measures, mesh word] | 1823 |
| 4 | 1 or 2 or 3 | 2950 |
| 5 | Qualifier*.mp. | 497 |
| 6 | Capacity.mp. | 112852 |
| 7 | Performance.mp. | 517775 |
| 8 | 5 or 6 or 7 | 612947 |
| 9 | 4 and 8 | 497 |
| 10 | limit 9 to yr="2024 -Current" | 30 |

# Scopus search (Literature Search performed: December 16, 2024)

| **Search name** | **Search query** | **Results** |
| --- | --- | --- |
| 10 | ( ( TITLE-ABS-KEY ( "International Classification of Functioning, Disability and Health" ) ) OR ( TITLE-ABS-KEY ( "International Classification of Functioning" ) ) OR ( TITLE-ABS-KEY ( icf ) ) ) AND ( ( TITLE-ABS-KEY ( qualifier* ) ) OR ( TITLE-ABS-KEY ( capacity ) ) OR ( TITLE-ABS-KEY ( performance ) ) ) AND PUBYEAR > 2000 AND PUBYEAR < 2026 | 2,668 |
| 9 | ( ( TITLE-ABS-KEY ( "International Classification of Functioning, Disability and Health" ) ) OR ( TITLE-ABS-KEY ( "International Classification of Functioning" ) ) OR ( TITLE-ABS-KEY ( icf ) ) ) AND ( ( TITLE-ABS-KEY ( qualifier* ) ) OR ( TITLE-ABS-KEY ( capacity ) ) OR ( TITLE-ABS-KEY ( performance ) ) ) | 2,845 |
| 8 | ( TITLE-ABS-KEY ( qualifier* ) ) OR ( TITLE-ABS-KEY ( capacity ) ) OR ( TITLE-ABS-KEY ( performance ) ) | 10,503,104 |
| 7 | TITLE-ABS-KEY ( performance ) | 8,476,008 |
| 6 | TITLE-ABS-KEY ( capacity ) | 2,551,694 |
| 5 | TITLE-ABS-KEY ( qualifier* ) | 2,808 |
| 4 | ( TITLE-ABS-KEY ( "International Classification of Functioning, Disability and Health" ) ) OR ( TITLE-ABS-KEY ( "International Classification of Functioning" ) ) OR ( TITLE-ABS-KEY ( icf ) ) | 15,895 |
| 3 | TITLE-ABS-KEY ( "International Classification of Functioning, Disability and Health" ) | 5,784 |
| 2 | TITLE-ABS-KEY ( "International Classification of Functioning" ) | 6,475 |
| 1 | TITLE-ABS-KEY ( icf ) | 13,724 |

## Supplementary literature search performed August 21, 2025 (Scopus, limited to 2024 – current).

| **Search name** | **Search query** | **Results** |
| --- | --- | --- |
| 10 | ( ( TITLE-ABS-KEY ( "International Classification of Functioning, Disability and Health" ) ) OR ( TITLE-ABS-KEY ( "International Classification of Functioning" ) ) OR ( TITLE-ABS-KEY ( icf ) ) ) AND ( ( TITLE-ABS-KEY ( qualifier* ) ) OR ( TITLE-ABS-KEY ( capacity ) ) OR ( TITLE-ABS-KEY ( performance ) ) ) AND PUBYEAR > 2023 AND PUBYEAR < 2026 | 349 |
| 9 | ( ( TITLE-ABS-KEY ( "International Classification of Functioning, Disability and Health" ) ) OR ( TITLE-ABS-KEY ( "International Classification of Functioning" ) ) OR ( TITLE-ABS-KEY ( icf ) ) ) AND ( ( TITLE-ABS-KEY ( qualifier* ) ) OR ( TITLE-ABS-KEY ( capacity ) ) OR ( TITLE-ABS-KEY ( performance ) ) ) | 3,003 |
| 8 | ( TITLE-ABS-KEY ( qualifier* ) ) OR ( TITLE-ABS-KEY ( capacity ) ) OR ( TITLE-ABS-KEY ( performance ) ) | 11,222,421 |
| 7 | TITLE-ABS-KEY ( performance ) | 9,088,682 |
| 6 | TITLE-ABS-KEY ( capacity ) | 2,707,609 |
| 5 | TITLE-ABS-KEY ( qualifier* ) | 2,898 |
| 4 | ( TITLE-ABS-KEY ( "International Classification of Functioning, Disability and Health" ) ) OR ( TITLE-ABS-KEY ( "International Classification of Functioning" ) ) OR ( TITLE-ABS-KEY ( icf ) ) | 16,621 |
| 3 | TITLE-ABS-KEY ( "International Classification of Functioning, Disability and Health" ) | 6,034 |
| 2 | TITLE-ABS-KEY ( "International Classification of Functioning" ) | 6,775 |
| 1 | TITLE-ABS-KEY ( icf ) | 14,358 |

# CINAHL via EBSCOhost (Literature Search performed: December 16, 2024)

| **#** | **Query** | **Limiters/Expanders** | **Last Run Via** | **Results** |
| --- | --- | --- | --- | --- |
| S11 | (S6 OR S7 OR S8) AND (S5 AND S9) | Limiters - Publication Date: 20010101-20241231 | Interface - EBSCOhost Research Databases | 887 |
|  |  | Expanders - Apply equivalent subjects | Search Screen - Advanced Search |  |
|  |  | Search modes - Proximity | Database - CINAHL |  |
| S10 | (S6 OR S7 OR S8) AND (S5 AND S9) | Expanders - Apply equivalent subjects | Interface - EBSCOhost Research Databases | 899 |
|  |  | Search modes - Proximity | Search Screen - Advanced Search |  |
|  |  |  | Database - CINAHL |  |
| S9 | S6 OR S7 OR S8 | Expanders - Apply equivalent subjects | Interface - EBSCOhost Research Databases | 413,7 |
|  |  | Search modes - Proximity | Search Screen - Advanced Search |  |
|  |  |  | Database - CINAHL |  |
| S8 | TX Performance | Expanders - Apply equivalent subjects | Interface - EBSCOhost Research Databases | 320,76 |
|  |  | Search modes - Proximity | Search Screen - Advanced Search |  |
|  |  |  | Database - CINAHL |  |
| S7 | TX Capacity | Expanders - Apply equivalent subjects | Interface - EBSCOhost Research Databases | 105,879 |
|  |  | Search modes - Proximity | Search Screen - Advanced Search |  |
|  |  |  | Database - CINAHL |  |
| S6 | TX Qualifier* | Expanders - Apply equivalent subjects | Interface - EBSCOhost Research Databases | 335 |
|  |  | Search modes - Proximity | Search Screen - Advanced Search |  |
|  |  |  | Database - CINAHL |  |
| S5 | S1 OR S2 OR S3 OR S4 | Expanders - Apply equivalent subjects | Interface - EBSCOhost Research Databases | 6,217 |
|  |  | Search modes - Proximity | Search Screen - Advanced Search |  |
|  |  |  | Database - CINAHL |  |
| S4 | TX ICF | Expanders - Apply equivalent subjects | Interface - EBSCOhost Research Databases | 4,244 |
|  |  | Search modes - Proximity | Search Screen - Advanced Search |  |
|  |  |  | Database - CINAHL |  |
| S3 | TX International Classification of Functioning | Expanders - Apply equivalent subjects | Interface - EBSCOhost Research Databases | 4,483 |
|  |  | Search modes - Proximity | Search Screen - Advanced Search |  |
|  |  |  | Database - CINAHL |  |
| S2 | TX (International Classification of Functioning, Disability, and Health) | Expanders - Apply equivalent subjects | Interface - EBSCOhost Research Databases | 4,337 |
|  |  | Search modes - Proximity | Search Screen - Advanced Search |  |
|  |  |  | Database - CINAHL |  |
| S1 | (MH "International Classification of Functioning, Disability, and Health") | Expanders - Apply equivalent subjects | Interface - EBSCOhost Research Databases | 3,031 |
|  |  | Search modes - Proximity | Search Screen - Advanced Search |  |
|  |  |  | Database - CINAHL |  |

## Supplementary literature search performed August 31, 2025 (CINAHL via EBSCOhost, limited to the last 12 months).

| **#** | **Query** | **Limiters/Expanders** | **Last Run Via** | **Results** |
| --- | --- | --- | --- | --- |
| S10 | (S6 OR S7 OR S8) AND (S5 AND S9) | Expanders - Apply equivalent subjects | Interface - EBSCOhost Research Databases | 319 |
|  |  | Search modes - Proximity | Search Screen - Advanced Search |  |
|  |  |  | Database - CINAHL |  |
| S9 | S6 OR S7 OR S8 | Expanders - Apply equivalent subjects | Interface - EBSCOhost Research Databases | 32,306 |
|  |  | Search modes - Proximity | Search Screen - Advanced Search |  |
|  |  |  | Database - CINAHL |  |
| S8 | TX Performance | Expanders - Apply equivalent subjects | Interface - EBSCOhost Research Databases | 24,887 |
|  |  | Search modes - Proximity | Search Screen - Advanced Search |  |
|  |  |  | Database - CINAHL |  |
| S7 | TX Capacity | Expanders - Apply equivalent subjects | Interface - EBSCOhost Research Databases | 10,730 |
|  |  | Search modes - Proximity | Search Screen - Advanced Search |  |
|  |  |  | Database - CINAHL |  |
| S6 | TX Qualifier* | Expanders - Apply equivalent subjects | Interface - EBSCOhost Research Databases | 52 |
|  |  | Search modes - Proximity | Search Screen - Advanced Search |  |
|  |  |  | Database - CINAHL |  |
| S5 | S1 OR S2 OR S3 OR S4 | Expanders - Apply equivalent subjects | Interface - EBSCOhost Research Databases | 643 |
|  |  | Search modes - Proximity | Search Screen - Advanced Search |  |
|  |  |  | Database - CINAHL |  |
| S4 | TX ICF | Expanders - Apply equivalent subjects | Interface - EBSCOhost Research Databases | 513 |
|  |  | Search modes - Proximity | Search Screen - Advanced Search |  |
|  |  |  | Database - CINAHL |  |
| S3 | TX International Classification of Functioning | Expanders - Apply equivalent subjects | Interface - EBSCOhost Research Databases | 403 |
|  |  | Search modes - Proximity | Search Screen - Advanced Search |  |
|  |  |  | Database - CINAHL |  |
| S2 | TX (International Classification of Functioning, Disability, and Health) | Expanders - Apply equivalent subjects | Interface - EBSCOhost Research Databases | 388 |
|  |  | Search modes - Proximity | Search Screen - Advanced Search |  |
|  |  |  | Database - CINAHL |  |
| S1 | (MH "International Classification of Functioning, Disability, and Health") | Expanders - Apply equivalent subjects | Interface - EBSCOhost Research Databases | 120 |
|  |  | Search modes - Proximity | Search Screen - Advanced Search |  |
|  |  |  | Database - CINAHL |  |

# Google Scholar Search (Literature Search performed: December 16, 2024)

Search String: ("ICF" OR "International Classification of Functioning" OR "International Classification of Functioning, Disability and Health") AND ("Qualifier*" OR "Capacity" OR "Performance")

Filters Applied:

Time period: 2001–[Current date].

Standard Google Scholar settings.

Procedure:

The first 200 hits out of approximately 140,000 results (0.04 seconds) were screened in order of relevance, as determined by Google Scholar's algorithm.

# Supplementary Grey Literature Search (Search performed: May 15, 2025)

| Source | Platform/Website | Search terms used | Number of results screened | Reports assessed for eligibility |
| --- | --- | --- | --- | --- |
| World Health Organization | [iris.who.int](https://iris.who.int/) | "International Classification of Functioning" AND qualifiers | 212 | 1 |
| World Health Organization | [iris.who.int](https://iris.who.int/) | "International Classification of Functioning" AND implementation | 405 | 2 |
| NICE (UK) | [nice.org.uk](https://www.nice.org.uk/) | "International Classification of Functioning" | 20 | Non |
| NICE (UK) | [nice.org.uk](https://www.nice.org.uk/) | ICF | 2 | Non |
| 1^st^ International Symposium on ICF Education | <https://icfeducation.org/category/events/previous-symposia/> | Keyword searches for “capacity,” “performance,” and “qualifiers” were conducted using the Ctrl+F function | Capacity: 7  Performance: 12  Qualifiers: 12 | Non |
| 2^nd^ International Symposium on ICF Education | Ditto | Ditto | Capacity: 6  Performance: 5  Qualifiers: 9 | 1 |
| 3^rd^ International Symposium on ICF Education | Ditto | Ditto | Capacity: 0  Performance: 0  Qualifiers: 0 | Non |
| 4^th^ International Symposium on ICF Education | Ditto | Ditto | Capacity: 12+13  Performance: 9+15  Qualifiers: 12+3 | Non |
| 5^th^ International Symposium on ICF Education | Ditto | Not possible to screen as it is only available in Russian. | *Not applicable* | *Not applicable* |
| 6^th^ International Symposium on ICF Education | Ditto, *could not be found* | *Not applicable* | *Not applicable* | *Not applicable* |
| 7^th^ International Symposium on ICF Education | <https://icfeducation.org/category/events/previous-symposia/> | Keyword searches for “capacity,” “performance,” and “qualifiers” were conducted using the Ctrl+F function | Capacity: 1  Performance: 1  Qualifiers: 5  NB: *Only a consensus statement was available.* | Non |
| 8^th^ International Symposium on ICF Education | Ditto, *could not be found* | *Not applicable* | *Not applicable* | *Not applicable* |
| 9^th^ International Symposium on ICF Education | Ditto | Ditto | Capacity: 10  Performance: 9  Qualifiers: 3 | 1 |

# Citation searching (Google Scholar “Cited by” Search performed: November 22, 2025)

| Study | Citation search type | Number of references screened | Reports assessed for eligibility |
| --- | --- | --- | --- |
| Costa et al., 2022 | Backward & Forward | Reference list (n = 30)  Google Scholar (n = 4) | 2 |
| Benito García et al., 2015 | Backward & Forward | Reference list (n = 37)  Google Scholar (n = 37) | 0 |
| Grill et al., 2007 | Backward & Forward | Reference list (n = 32)  Google Scholar (n = 71) | 2 |
| Caporaso et al., 2023 | Backward & Forward | Reference list (n = 9)  Google Scholar (n = 2) | 4 |
| Dalavina et al., 2025 | Backward & Forward | Reference list (n = 26)  Google Scholar (n = 0) | 3 |
