# Supplementary material for: Exploring clinician-reported assessments of capacity and performance qualifiers in the ICF: a scoping review
Source: Front Rehabil Sci. 2026 May 7;7:1792865. doi: 10.3389/fresc.2026.1792865 (PMC13191736; doi:10.3389/fresc.2026.1792865)
Supplement: Supplementary file 2 [file Supplementaryfile2.docx]

Supplementary File 2: Interrater Agreement

Table S1. Interrater agreement during title and abstract screening

| Reviewer A | Reviewer B | A Include / B Include | A Include / B Exclude | A Exclude / B Include | A Exclude / B Exclude | Observed agreement | Expected agreement | Cohen’s κ |
| --- | --- | --- | --- | --- | --- | --- | --- | --- |
| Kristian Hansen | Tobias Kaarsbo | 22 | 20 | 17 | 1602 | 0.978 | 0.952 | 0.532 |
| Thomas Maribo | Tobias Kaarsbo | 26 | 18 | 87 | 420 | 0.809 | 0.748 | 0.244 |
| Jeppe Phillip | Tobias Kaarsbo | 117 | 93 | 64 | 1820 | 0.925 | 0.831 | 0.557 |

Table S2. Interrater agreement during full-text screening

| Reviewer A | Reviewer B | A Include / B Include | A Include / B Exclude | A Exclude / B Include | A Exclude / B Exclude | Observed agreement | Expected agreement | Cohen’s κ |
| --- | --- | --- | --- | --- | --- | --- | --- | --- |
| Jeppe Phillip | Tobias Kaarsbo | 2 | 0 | 0 | 142 | 1.000 | 0.973 | 1.000 |
| Kristian Hansen | Tobias Kaarsbo | 1 | 2 | 1 | 106 | 0.973 | 0.956 | 0.387 |
| Thomas Maribo | Tobias Kaarsbo | 1 | 0 | 0 | 0 | 1.000 | 1.000 | NA |
